# BIRC5 promotes cancer progression and predicts prognosis in laryngeal squamous cell carcinoma

**DOI:** 10.7717/peerj.12871

**Published:** 2022-02-01

**Authors:** Nan Wang, Xuanyu Huang, Jinsheng Cheng

**Affiliations:** 1 Guangdong Provincial Key Laboratory of Conservation and Precision Utilization of Characteristic Agricultural Resources in Mountains Areas, School of Life Science of Jiaying University, Meizhou, China; 2 Henry-Fork School of Food Sciences, Shaoguan University, Shaoguan, China

**Keywords:** Laryngeal squamous cell carcinoma, Bioinformatics, BIRC5, Prognosis, Cell apoptosis

## Abstract

**Background:**

Laryngeal squamous cell carcinoma (LSCC) remains one of the most common respiratory tumors worldwide. Baculoviral inhibitor of apoptosis repeat containing 5 (*BIRC5)* is a member of the inhibitor-of-apoptosis protein family. BIRC5 plays an important role in various types of cell proliferation, differentiation, migration and invasion. However, the specific role of BIRC5 in LSCC remains unclear.

**Methods:**

To provide a prognostic biomarker for LSCC, we screened the prognostic genes of LSCC *via* bioinformatics. PPI network and KEGG pathways were used to select hub genes. Clinical prognoses were performed using a Kaplan–Meier plotter and Cox proportional-hazard analysis. BIRC5 expression in LSCC tissues and cell lines were detected by RT-PCR, Western blot and Immunohistochemistry (IHC). Cell proliferation, cell cycle and cell apoptosis were detected with Cell Counting Kit-8 (CCK-8) and Flow Cytometry assay, respectively.

**Results:**

Here, *BIRC5* was strongly correlated with higher tumor grade and differentiation. BIRC5 was highly expressed in LSCC tissues when compared with normal tissues and increased expression of BIRC5 was associated with overall survival in LSCC patients. The suppression of BIRC5 induced cell apoptosis and cell cycle arrest, thereby inhibiting the proliferation of LSCC cells. The survival analysis confirmed that higher level of BIRC5 expression predicted poor prognosis of LSCC patients. BIRC5 may act as an oncogene of LSCC development and was suggested as a promising prognostic biomarker for LSCC.

## Introduction

Laryngeal squamous cell carcinoma (LSCC) is the second most common form of head and neck carcinoma (HNC) with high incidence rate (177,422 new cases) in 2018 (Bray et al., 2018). LSCC includes malignant squamous lesions in the larynx, maxillofacial and pharynx, of which more than 90% are squamous cell carcinoma. Currently, the occurrence of laryngeal carcinoma in China is linearly increasing and approximately four times higher than in the United States (Torre et al., 2015). Despite considerable advances and progress in standard treatment, the 5-year survival rate of LSCC patients with advanced stages remains dismal. The 5-year survival rate of patients with early stages are more than 80%, but only 30–40% for patients with lymph node metastasis, and less than 20% of patients with distant metastasis (Ferlito et al., 2001; Kalavrezos & Bhandari, 2010). Unfortunately, most of the LSCC patients are diagnosed in advanced stages. So far, the clinical diagnosis, treatment and prognosis of patients are formulated and determined based on TNM stage, LSCC lacks a sensitives diagnosis marker at an early stage and to predict the prognosis. Therefore, it is of far-reaching significance to gradually establish effective personalized evaluation indexes and evaluation system.

Baculoviral inhibitor of apoptosis repeat containing 5 (*BIRC5*), a mitotic spindle checkpoint gene, which has been shown to display vital roles in cell proliferation, differentiation, migration and invasion (Coumar et al., 2013; Wang et al., 2012; Yamamoto, Ngan & Monden, 2008; Zwerts et al., 2007). Previous studies indicated that BIRC5 promotes mitosis through formation of the chromosomal passenger complex (CPC) with CDCA8 and INCENP, and regulates the microtubule dynamics during G2/M phase (Rosa et al., 2006). On the other hand, BIRC5 is elevated in some solid tumors and associated with poor prognosis (Lv et al., 2010). Down-regulation of BIRC5 decreased the proliferation of triple-negative breast cancer cells, implying that BIRC5 acts as a tumor driver (Wang et al., 2012). The association between BIRC5 and LSCC remains limited. Although it was reported that BIRC5 was associated with therapy resistance in head and neck cancer patients (Knauer et al., 2013). However, the specific roles of BIRC5 and mechanisms in the carcinogenesis of LSCC remains to be elucidated.

Therefore, in the present work, we evaluated the significance of BIRC5 gene expression pattern, prognostic value and protein interaction network in LSCC by using comprehensive bioinformatics analysis in several large online databases. These findings uncover the important role of BIRC5 in LSCC as well as define a potential therapeutic target for LSCC.

## Materials and Methods

### Cell culture and human tissues

TU_211-CVCL 4915 and TU_211-CVCL 4916 cell lines were obtained from Guangzhou Juyan Biological Technology (Guangzhou, China) and cultured in RPMI 1640 (Gibco, Waltham, MA, USA). Cell culture media were supplemented with 10% FBS (Gibco, Waltham, MA, USA) and 1% penicillin–streptomycin solution (Invitrogen, Waltham, MA, USA). Prior to infection or/and transfection, all cells were grown in 6-well/96-well plates for 0–96 h. The cells were grown in a humidified atmosphere of 5% CO_2_ at 37 °C and tested negative for mycoplasma contamination. A total of 88 paired formalin-fixed, paraffin-embedded tissues were collected from the medical school of Jiaying University. This study was conducted in accordance with the Helsinki Declaration and informed consent was obtained from patients. The study was approved by the Ethics Committee of Jiaying University.

### Data acquisition

The GEPIA (http://gepia.cancer-pku.cn/index.html) was used to confirm the relative levels of gene expression in normal and tumor samples. The selection criteria are as follows: Log_2_FC cutoff: 1, *P*-value cutoff: 0.01. The Ualcan (http://ualcan.path.uab.edu/) was used to validate the relative level of *BIRC5* in tumor subgroups according to different grades (Chandrashekar et al., 2017). Genes were pasted into the text and chose the cancer type. The relative expression level and survival information of the gene in normal *vs*. tumor samples and across cancer subgroups were output.

### RNA isolation and real-time PCR

Total RNA was extracted using the TRIzol reagent (Invitrogen, Waltham, MA, USA). One µg of RNA from each sample was reverse transcribed into cDNA using the HiScript III 1st Strand cDNA Synthesis Kit (Vazyme, Nanjing, China). PCR amplification was performed using SYBR green kit on a Light Cycler 480 (Roche, Basel, Switzerland). Relative RNA expression data was normalized to housekeeping gene GAPDH and calculated using the 2^–ΔΔCT^ method (Livak & Schmittgen, 2001). The specific primer sequence were as follows: *BIRC5* forward, 5′ ATTCGTCCGGTTGCGCTTTCC 3′; reverse, 5′ CACGGCGCACTTTCTTCGCAG 3′; and *GAPDH* forward, 5′ GCACCACCAACTGCTTAGCA 3′; reverse, 5′ TCTTCTGGGTGGCAGTGATG 3′.

### Kaplan–Meier plotter database analysis

The Kaplan–Meier Plotter Database (http://kmplot.com/analysis/) was used to analyze the correlation between BIRC5 expression and survival in head and neck squamous cancer (HNSC). We analyzed the overall survival of HNSC patients by using a Kaplan–Meier survival plot. First, chose Pan-cancer RNA-seq, then genes were uploaded into the database, and the inclusion criteria was: Head-neck squamous cell carcinoma (*n* = 500), Follow up threshold: 240 months, and number-at-risk were also shown. The hazard ratio (HR) with 95% confidence intervals and long rank *P*-value were calculated and displayed on the webpage.

### Microarrays in GEO datasets and profiles

The Gene Expression Omnibus (http://www.ncbi.nlm.nih.gov/geo) is an open database of microarray/gene profiles (Barrett et al., 2011). In order to understand the diagnostic value of BIRC5 in LSCC, two mRNA expression datasets containing microarray data from normal tissues and LSCC tissues, GSE51985 (Lian et al., 2013) and GSE59102 (Placa et al., 2015), were selected. The GSE51985 microarray data including 10 adjacent non-neoplastic tissues and 10 LSCC tissues and were generated using Illumina HumanHT-12 V4.0 expression beadchip. The GSE59102 microarray data including 13 normal tissues and 29 LSCC tissues and were generated using Agilent-014850 Whole Human Genome Microarray 4 × 44K G4112F.

### Cell cycle analysis

Cells were grown in six-well plates until they achieved 30–40% confluence. After transfected with shBIRC5 or shNC for 48 h, the cells were fixed with 75% ethanol at 4 °C overnight, then centrifuged at 300×*g* for 5 min. RNase A (50 µg/ml) and propidium iodide (50 µg/ml) (Sigma, St. Louis, MO, USA) were added into cells at room temperature for 15 min. Cell cycle was analyzed by BD Accuri^TM^ C6 flow cytometry (BD Biosciences, Franklin Lakes, NJ, USA) according to the manufacturer’s protocol and determined by ModFit LT 4.1 software (BD Biosciences, Franklin Lakes, NJ, USA), CXP software was used to determine the cell percentage in different phases. The linearity was set to two and internal standards were also selected.

### Cell proliferation and colony-formation assays

Cells were seeded in 96-well plates at 2 × 10^3^ per well for 24 h. Cell proliferation was evaluated using CCK8 Assay kit according to the manufacturer’s instructions. CCK8 reagent was added to cell culture medium after 24, 48, 72, and 96 h, and incubated for 2 h. The absorbance at 490 nm was measured using a Spark^®^ multimode microplate reader (Tecan, Männedorf, Switzerland). Each experiment was repeated three times. For colony-formation assays, 500 cells were seeded in six-well plate and incubated in complete growth medium for 7–14 days. Cell colonies were fixed with methanol and stained with 0.1% crystal violet (Sigma-Aldrich, St. Louis, MO, USA) in order to be counted manually.

### Bioinformatics analysis

To screen the DEGs between LSCC tissues and normal tissues, two LSCC gene expression series were analyzed by GEO2R (http://www.ncbi.nlm.nih.gov/geo/geo2r/). The cutoff value criteria were: *P* value < 0.05 and |log_2_FC| > 2. For the overlapping genes, the DAVID database (http://david.ncifcrf.gov/) was used to perform GO enrichment and KEGG pathway analysis (Huang, Sherman & Lempicki, 2009). Venn diagram (http://bioinformatics.psb.ugent.be/webtools/Venn/) was used to calculate the intersections of the list of elements. Further, we used mcode to analyse the GO enrichment pathway including biological process, cellular component and molecular function (Shannon et al., 2003). Meanwhile, the STRING database (http://string-db.org/) was designed to provide a protein–protein interaction network analysis (Szklarczyk et al., 2015). Hub genes in LSCC were obtained from the network. Univariable and multivariable Cox regression were performed to analyses survival-associated genes.

### Western blot

The cells were transfected with shBIRC5 and shNC for 48 h and harvested with lysis buffer (Solarbio, Beijing, China) including protease inhibitors (Roche, Basel, Switzerland). Overall, 30 µg total protein was separated by SDS-PAGE and transferred to PVDF membranes (Millipore, Burlington, MA, USA) and blocked with 5% bovine serum albumin, then the membranes were incubated with primary antibodies overnight at 4 °C. Immunoblots were detected using ECL detection reagent (Thermo Fisher Scientific, Waltham, MA, USA) according to the manufacturer’s protocol. Antibodies used were: BIRC5 (sc-17779; Santa Cruz), GAPDH (#5174; Cell Signaling Technology).

### Lentivirus transduction

The lentiviral shRNA plasmid targeted BIRC5 were from Ribo Bio Company (Guangzhou, China). The BIRC5 shRNA sequence was: CCGGCCTTTCTGTCAAGAAGCAGTTCTCGAGAACTGCTTCTTGACAGAAAGGTTTTTG. Briefly, the recombinant lentivirus was harvested by co-transfecting 293T cells. After an additional 72 h, culture media were collected and the aliquot supernatant was stocked at −80 °C for infection in future. The efficiency of BIRC5 knockdown were evaluated by qPCR and western blot.

### Immunohistochemistry (IHC) and antibodies

The Human Protein Atlas (HPA) is an online antibody-based proteomics database of human disease and normal tissues (https://www.proteinatlas.org/). In this study, representative BIRC5 expression was obtained from HPA in normal tissues and LSCC tissues to demonstrate the potential expression pattern in clinical practice. Our cohort tissues from 88 cases of LSCC fixed in the formalin and embedded in the paraffin for BIRC5 immunohistochemistry staining. BIRC5 antibody for IHC was obtained from Santa Cruz (1:1,000, sc-17779).

### Statistical analysis

Stata 12.0 was used to perform a GEO microarray meta-analysis in this study. Statistical analyses were performed using GraphPad Prism 7.0a and SPSS 20.0 software. Two-tailed Student’s *t*-test or ANOVA were used to compare experimental groups as indicated. Cox proportional hazards regression model were used for multivariable analyses. The data were presented as mean ± SD. *P* < 0.05 was considered to be statistically significant.

## Results

### Bioinformatics analysis of the DEGs in LSCC

**Figure 1:**
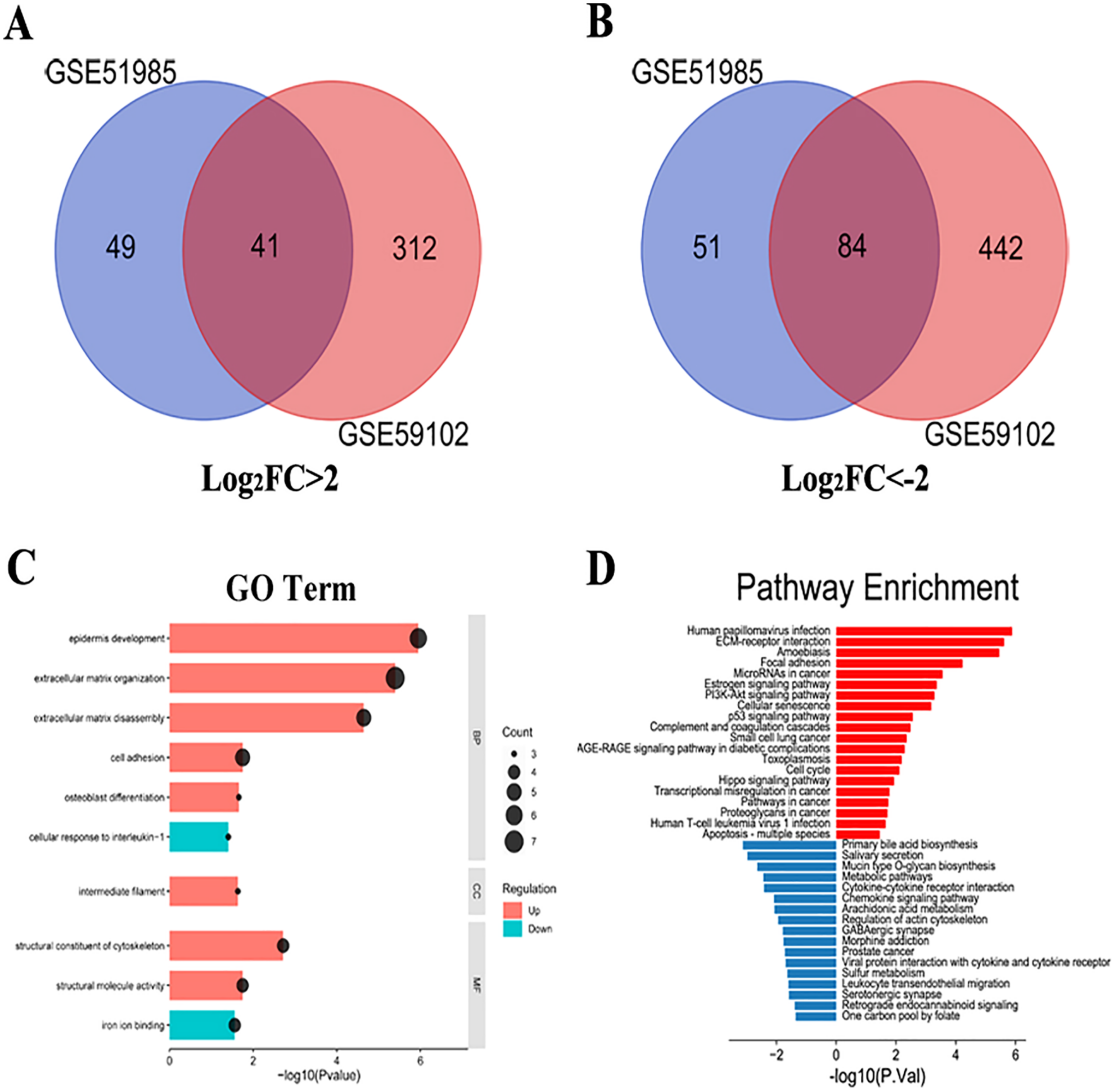
LSCC DEGs screening by bioinformatics. (A) A total of 41 upregulated LSCC DEGs were screened from the GSE51985 and GSE59102 profiles. (B) A total of 84 downregulated LSCC DEGs were screened from the GSE51985 and GSE59102 profiles. (C) GO and (D) KEGG analysis of significantly DEGs between normal and LSCC tissues.

A total of 1,287 LSCC DEGs were screened from two gene expression profiles (GSE51985 and GSE59102) in the GEO database. Then, the upregulated and downregulated DEGs of GSE51985 and GSE59102 showing an overlap region (Figs. 1A and 1B). Moreover, compared with normal tissues, 41 DEGs with absolute *P* < 0.05 and log_2_FC > 2 were deemed to be upregulated and 84 DEGs with *P* < 0.05, log_2_FC < –2 were downregulated in LSCC tissues (Tables S1 and S2). The DEGs enrichment of GO terms (biological process, molecular function and cell component) and KEGG pathways analysis were performed by DAVID. Fig. 1C indicates the top 10 highly enriched GO items and the significantly enriched KEGG pathways were shown in Fig. 1D. The DEGs were enriched in the Human papillomavirus infection, cellular senescence, cell cycle and pathways in cancer.

### PPI network analysis and KEGG reanalysis

**Figure 2:**
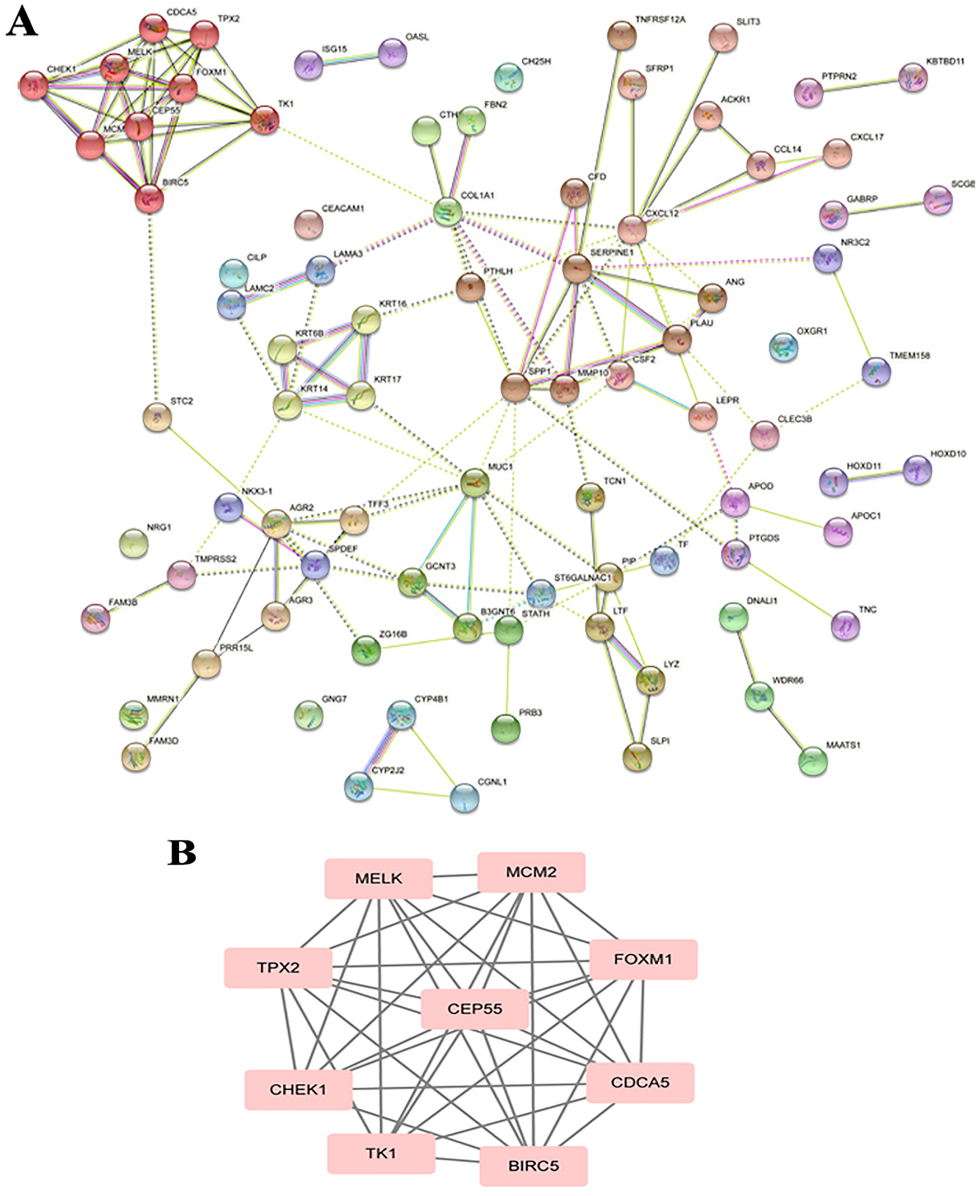
STRING analysis DEG PPI network. (A) Protein–protein network of 157 DEGs in LSCC was produced by the STRING online server. Pink rectangles indicated the up-regulated genes, green rectangles indicated down-regulated genes. (B) Nine hub genes obtained from the PPI network. A degree >10 was chosen as the cut-off.

**Figure 3:**
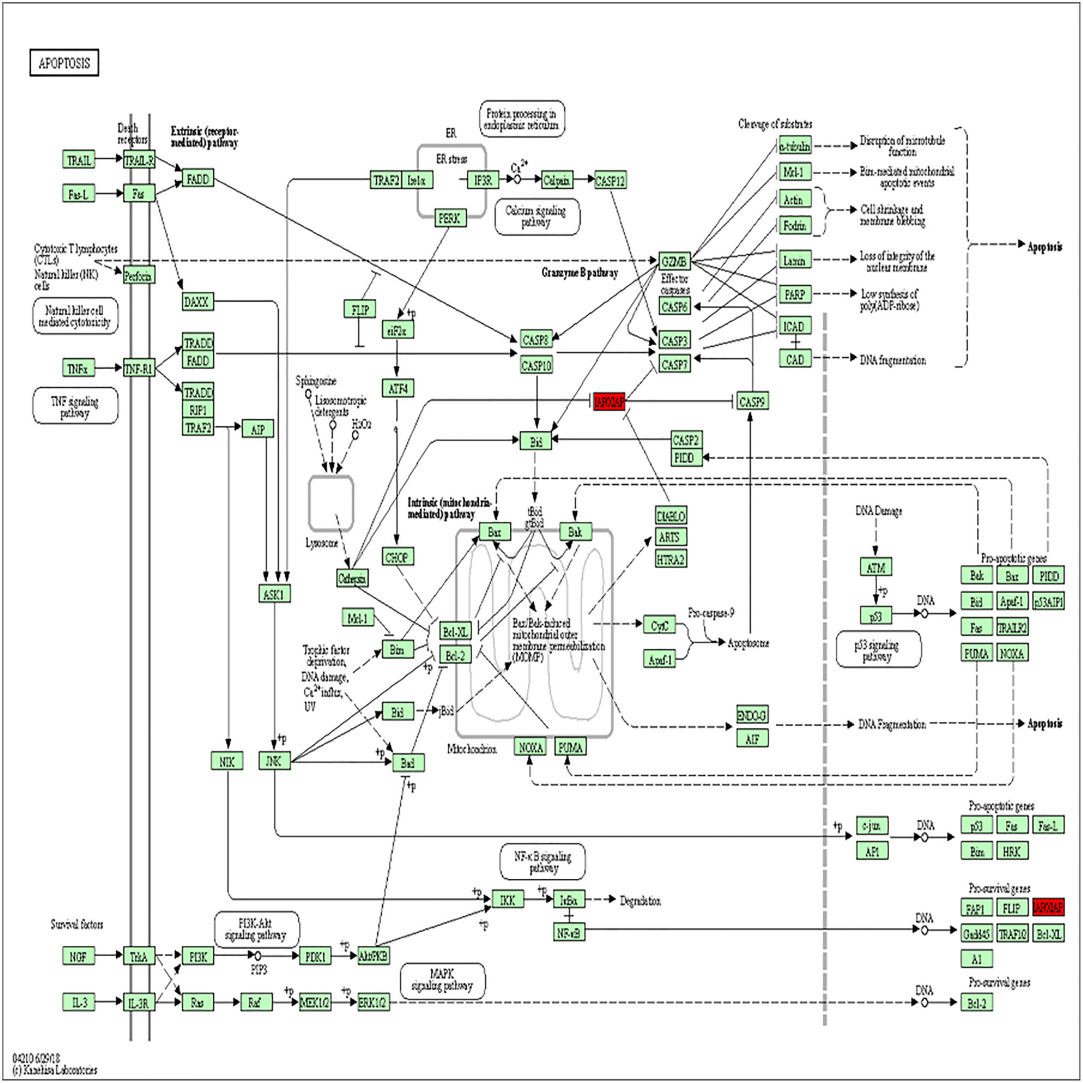
BIRC5 was significantly enriched in the apoptosis pathway. BIRC5 was significantly enriched in the apoptosis pathway. Previous HGNC symbols for BIRC5 gene was IAP4.

The PPI analysis among DEGs was constructed by STRING database. The network displayed 79 upregulated genes and 78 downregulated genes (Fig. 2A). These genes were further visualized by Cytoscape MCODE, of which *MELK*, *MCM2*, *TPX2*, *FOXM1*, *CEP55*, *CHEK1*, *CDCA5*, *TK1* and *BIRC5* genes were all upregulated (Fig. 2B and Table 1). DAVID was applied to reanalysis the biological functions of the above nine DEGs. Additionally, 3 out of 9 genes associated with the apoptosis pathway (Fig. 3).

**Table 1:** All DEGs were detected from two mRNA expression GEO datasets.

Gene	MCODE_Score	Degree	Expression level	GSE51985 *P* value	GSE59102 *P* value
BIRC5	6.8	9	Up	6.12E−04	2.61E−09
CEP55	6.8	9	Up	4.95E−03	6.01E−10
CHEK1	7.0	7	Up	1.66E−03	1.29E−11
TPX2	6.8	8	Up	2.51E−04	1.93E−11
FOXM1	6.8	11	Up	1.63E−04	6.43E−10
CDCA5	6.8	8	Up	2.26E−04	2.17E−11
MELK	6.8	8	Up	1.25E−03	4.88E−09
MCM2	6.8	8	Up	5.60E−05	1.48E−14
TK1	7.0	8	Up	5.09E−04	1.20E−12

### Core genes expression and prognosis in LSCC

**Figure 4:**
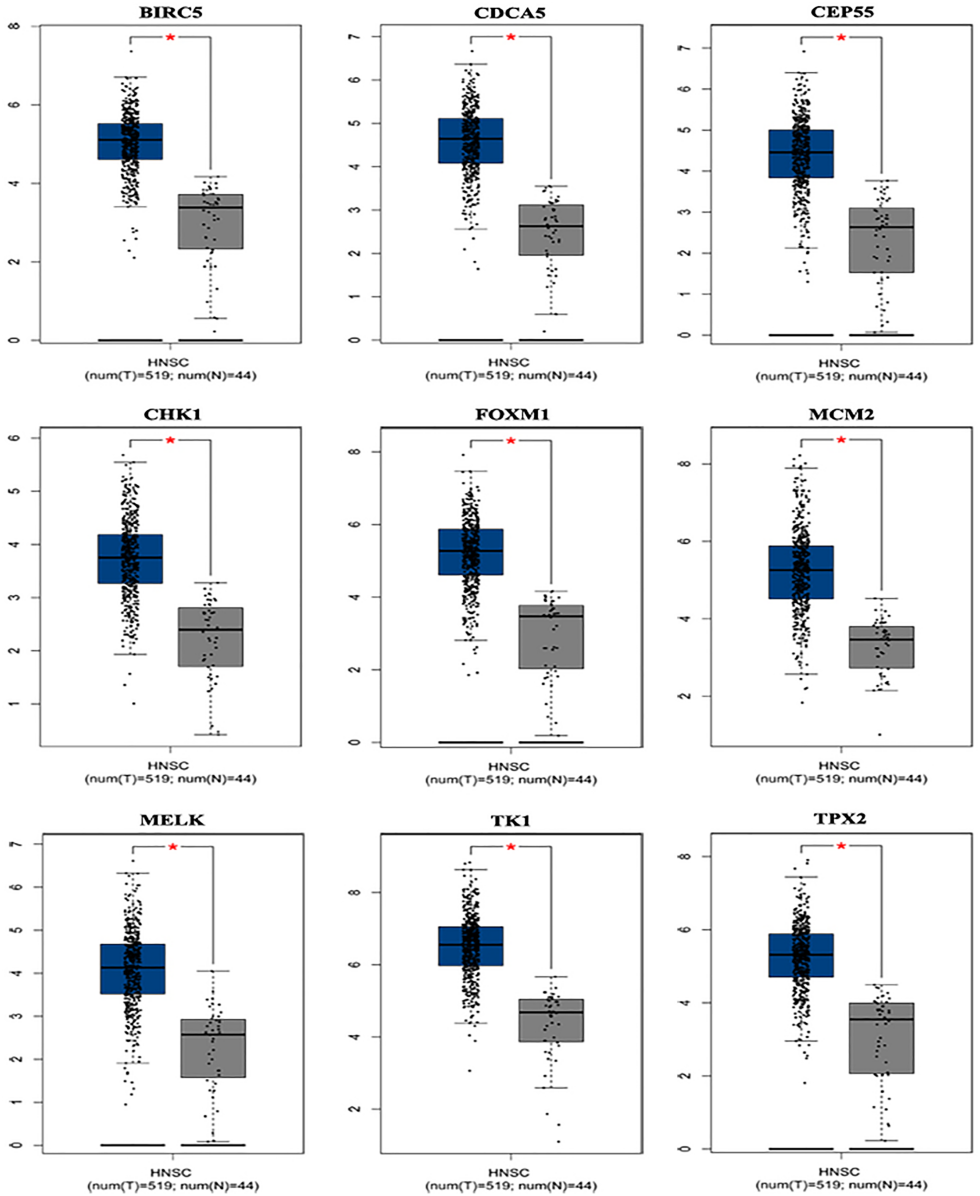
Differential expression analysis of nine genes mRNA in head and neck squamous cell cancer patients. Box plot evaluating gene expression in normal and LSCC patients obtained from the GEPIA database.*P < 0.01.

**Figure 5:**
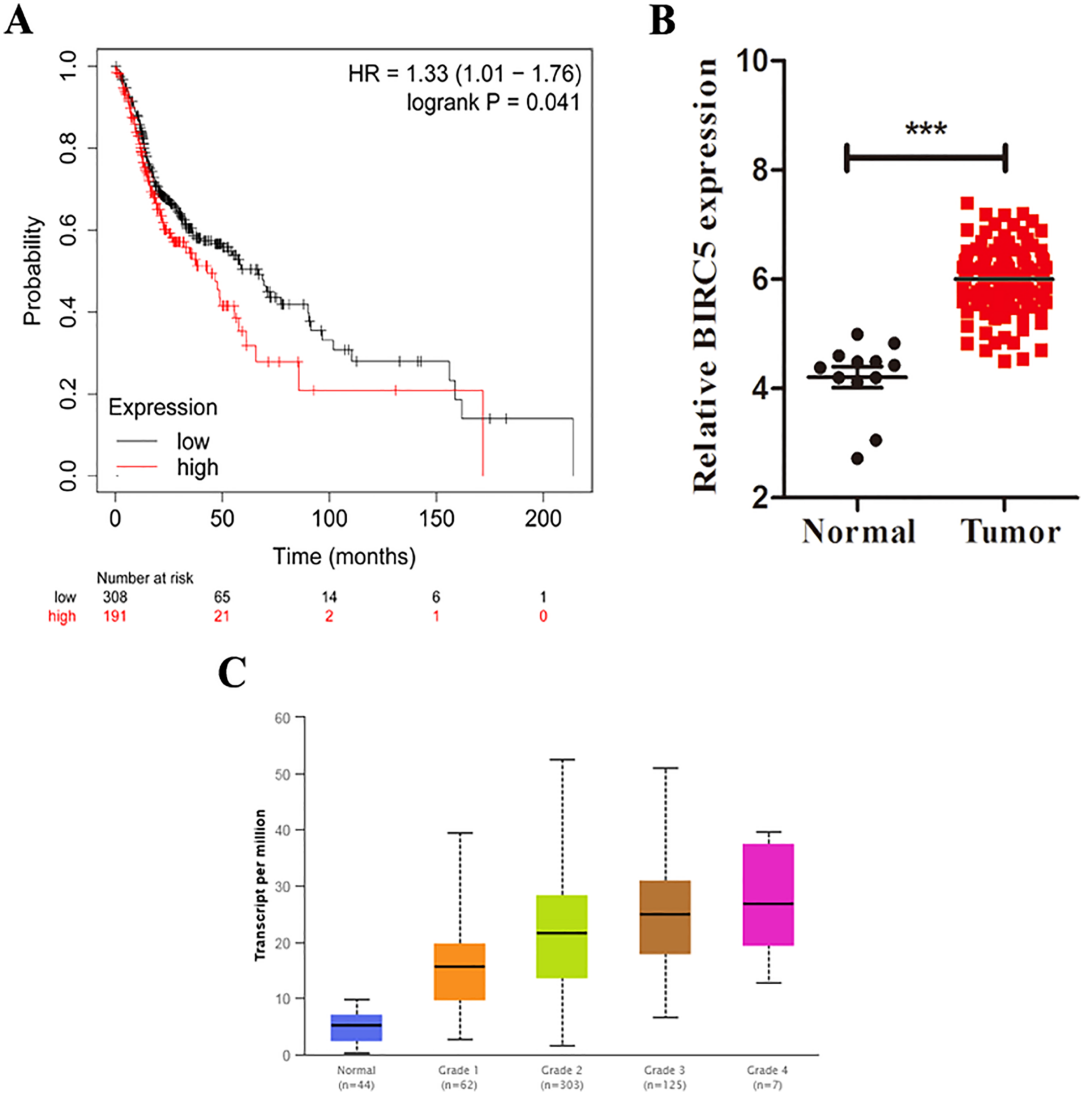
BIRC5 is associated with prognosis and tumor grades of LSCC patients. (A) Kaplan–Meier survival curve of LSCC patients with different BIRC5 level in the TCGA cohort. (B) Relative BIRC5 expression in TCGA-LSCC tissues. (C) BIRC5 mRNA expression in primary LSCC tissues from patients with different tumor grades from UALCAN. ***p < 0.001.

Based on the TCGA and GTEx, the level of core genes expression in normal and LSCC patients were validated by GEPIA. Fig. 4 indicated the expression of nine core genes in LSCC patients and normal patients. Then, we investigated the prognostic value of core genes using Kaplan–Meier analysis. As shown in Fig. 5A, among the above genes only high *BIRC5* showed shorter overall survival when compared with other genes. Further, we evaluated BIRC5 expression in LSCC by using the RNA-seq data in TCGA (Fig. 5B). By mining previously reported data, we found that high level of *BIRC5* was significantly associated with higher tumor grade (Fig. 5C).

### BIRC5 is overexpressed in LSCC patients

**Figure 6:**
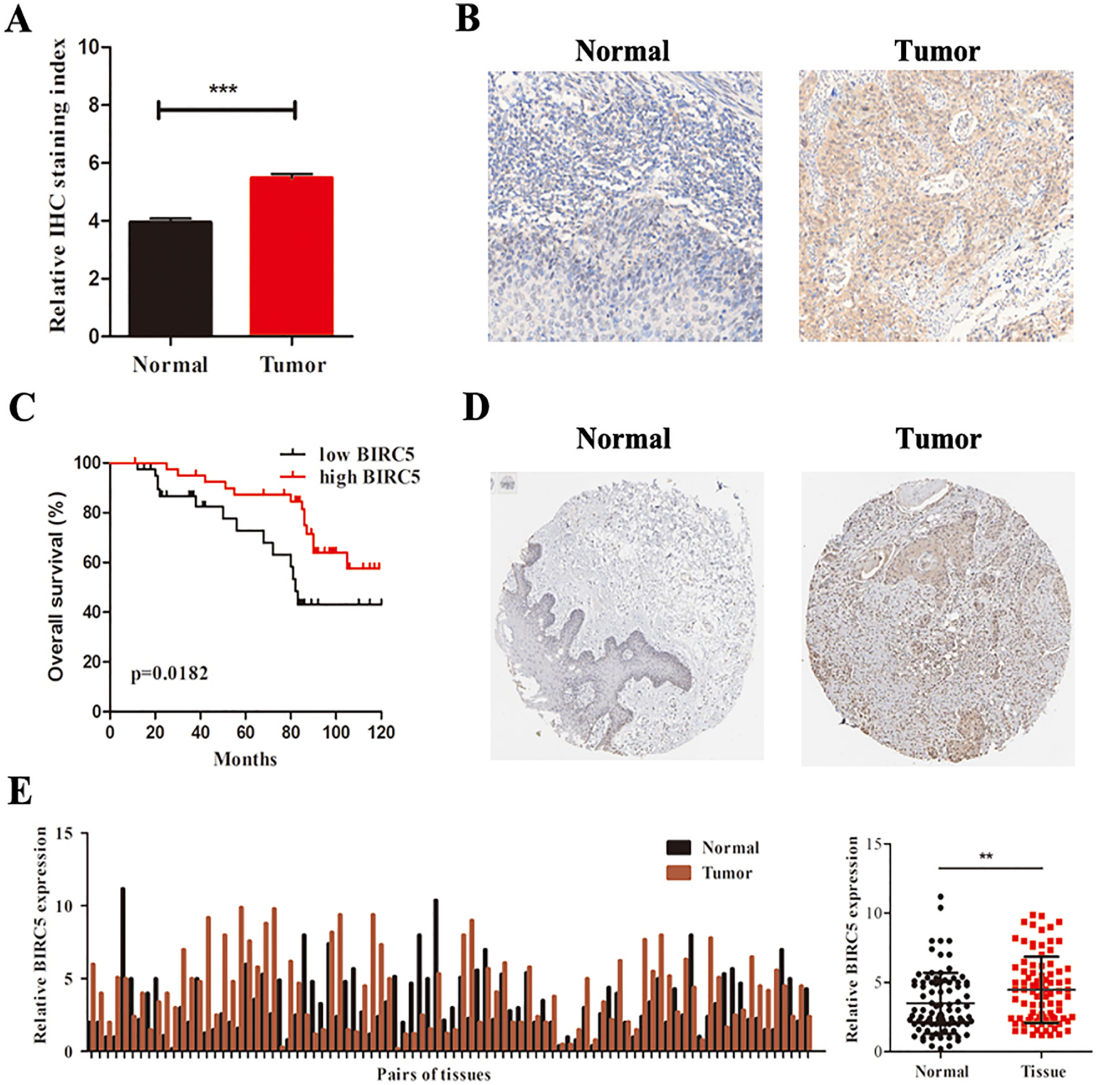
BIRC5 is overexpressed in LSCC patients. (A) BIRC5 expression was significantly higher in LSCC tissues compared with that in the adjacent normal tissues. (B) Representative IHC staining of BIRC5, Scale bar, 200 μm. (C) Overall survival estimates of 88 LSCC patients according to BIRC5 expression. (D) Representation of IHC staining of normal oral mucosa and laryngeal squanous carcinoma from HPA using anti-BIRC5. Scale bar, 100 μm. (E) Relative expression of BIRC5 in 88 LSCC patients. **P < 0.01, ***P < 0.001.

To further determine the expression level of BIRC5 in LSCC patients, we analyzed 88 primary LSCC patients and matched adjacent normal tissues by immunochemistry in our cohort. The histological types of LSCC were determined according to the World Health Organization system. BIRC5 was significantly upregulated in 88 LSCC tissues (Figs. 6A and 6B). Kaplan–Meier survival analysis of our cohort showed that higher BIRC5 patients had shorter overall survival, which was consistent with the online database results (Fig. 6C). We then validated the protein level of BIRC5 from HPA and found BIRC5 was also increased in LSCC tissues (Fig. 6D). In addition, RT-PCR analysis showed that the expression level of BIRC5 in LSCC was higher than that of matched adjacent normal tissues (Fig. 6E). These findings suggested a positive correlation between BIRC5 upregulation and LSCC progression.

### BIRC5 serves as an independent prognostic marker in LSCC patients

To explore the relationship between BIRC5 and LSCC clinicopathologic parameters, we analyzed the corresponding clinical data in 88 pairs of LSCC tissues. The clinical characteristics were showed in Table 2. The results indicated that BIRC5 expression was related with advanced tumor grade and differentiation. Then, we performed univariable and multivariable cox regression analysis to select independent clinicopathological prognostic factors for LSCC from TCGA cohort. Multivariable analysis indicated that BIRC5 expression and differentiation grade were independent factors for OS (Table 3). Taken together, BIRC5 can serve as an independent prognostic factor in patients with LSCC.

**Table 2:** Correlation between BIRC5 expressions with clinicopathological characteristics of LSCC.

Characteristics	*n*	BIRC5 expression		*P*
		High (56)	Low (32)	
Age(years)				0.920
≤50	32	15	17	
>50	56	41	15	
Smoking status				0.186
Yes	70	25	45	
No	10	4	6	
Alcohol abuse				0.061
Never	13	6	7	
Ever	75	34	41	
Tumor grade				0.000
I	23	5	18	
II	18	11	7	
III/IV	47	40	7	
Tumor stage				0.308
T_1_/T_2_	55	35	20	
T_3_/T_4_	33	21	12	
Lymph node metastasis				0.823
N0.N1	42	18	24	
N3.N4	46	38	8	
Differentiation				0.047
Well	51	37	14	
Moderate-poor	37	19	18	

**Table 3:** Univariate and multivariate Cox regression analysis results from the TCGA-LSCC cohort.

Variables	Univariate analysis		Multivariate analysis	
	HR 95% CI	*P*	HR 95% CI	*P*
Age (≥50 *vs*. <50]	0.639 [0.261–1.565]	0.327		
Smoking status	0.717 [1.140–2.141]	0.173		
Alcohol abuse	1.142 [0.871–1.490]	0.092		
Tumor grade (N1.2 *vs*. N3.4)	1.487 [1.346–3.733]	0.016		
Tumor stage (T1.2 *vs*. T3.4)	0.795 [0.340–1.157]	0.196		
Lymph node metastasis	0.209 [0.021–20.19]	0.139		
**(**N1.2 *vs*. N3.4)				
TNM stage (Late *vs*. Early)	1.296 [1.346–1.733]	0.081		
Differentiation (Well *vs*. Poor)	1.862 [1.771–2.255]	<0.001	1.792 [1.086–4.048]	<0.001
BIRC5 expression (High *vs*. Low)	1.504 [1.346–1.733]	**<**0.001	3.204 [1.740–5.017]	<0.001

### BIRC5 promotes cell proliferation and controls cells cycle and apoptosis *in vitro*

**Figure 7:**
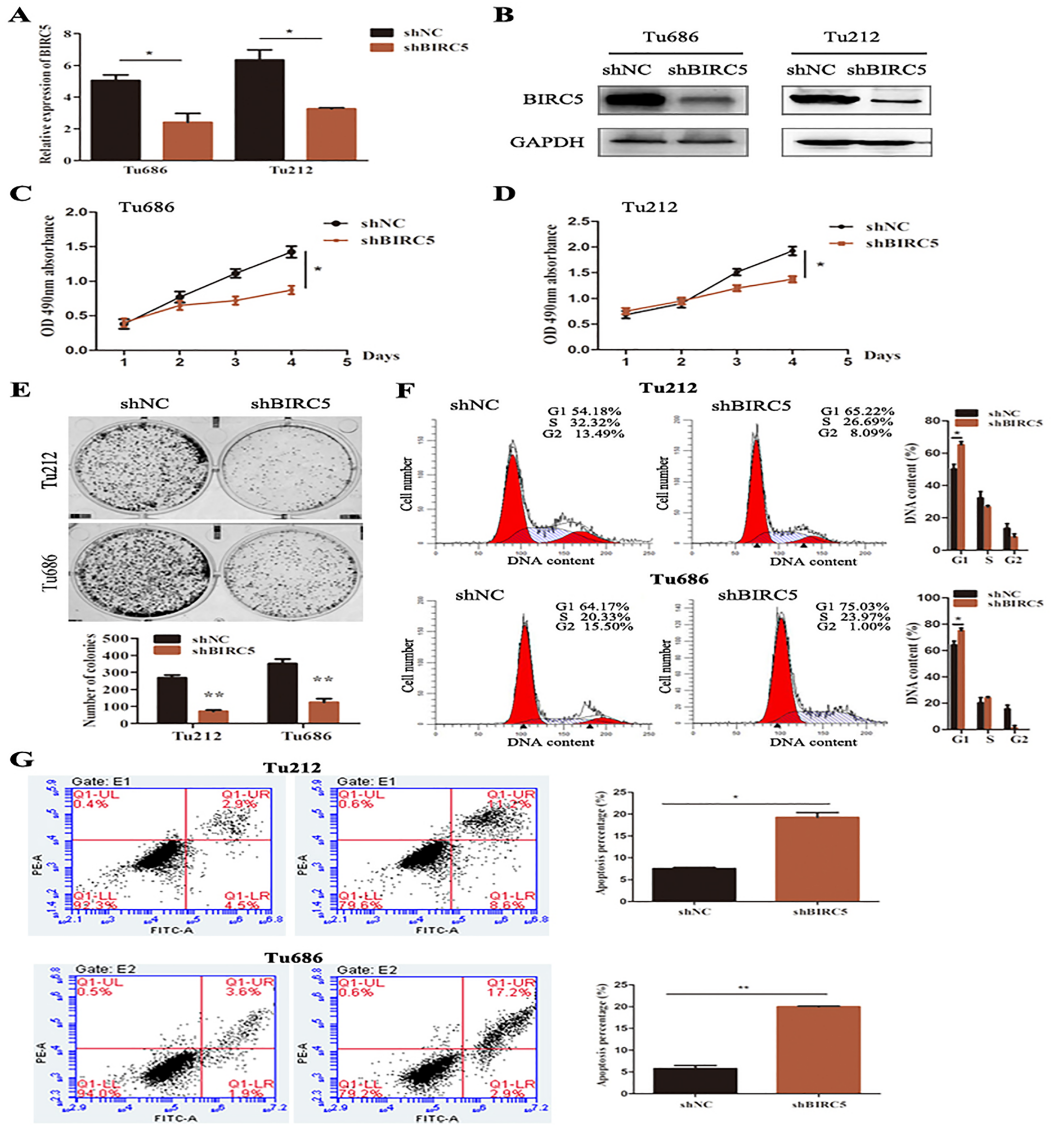
Epigenetic silencing of BIRC5 inhibits proliferation of LSCC cells and controls cell cycle progression and apoptosis *in vitro*. (A) mRNA levels of BIRC5 in indicated cells transfected with Negative Control (NC) shRNA and BIRC5 shRNA. (B) Protein levels of BIRC5 in indicated cells with shRNA and shBIRC5. BIRC5-specific shRNA significantly decreased the proliferation of Tu212 (C) and Tu686 (D) cells shown by CCK8 assay. (E) Colony formation assays were performed in BIRC5 inhibiting Tu686 and Tu212 cells. (F) Representative images and quantification of Negative Control (NC) shRNA and BIRC5 shRNA-transfected Tu212 and Tu686 cells were analyzed by cell cycle assay. (G) Representative images and quantification of cell apoptosis assay. *P < 0.05, **P < 0.01.

To further verify the above bioinformatics analysis, BIRC5-specific shRNA was exploited to detect with RT-PCR and western blot analysis. BIRC5 was effectively knocked down in Tu212 and Tu686 cells (Figs. 7A and 7B). To investigate the role of BIRC5 on LSCC cell proliferation, CCK8 assay and colony formation assay were performed. CCK8 assay revealed that the proliferation ability was reduced after BIRC5 depletion (Figs. 7C and 7D). Silencing BIRC5 significantly inhibited the colony-forming capabilities of Tu212 and Tu686 cells (Fig. 7E). Next, we further explored the functional impact of BIRC5 expression on cell growth behavior of LSCC cells. Flow cytometry was performed to analyze the changes in cell cycle, as shown in Fig. 7F, cells with BIRC5 suppression revealed that cell cycle was arrest in G1 phase, confirming the role of BIRC5 in controlling cell cycle progression. In addition, the percentage of apoptotic cells in the shBIRC5-transfected group was shown higher than the shNC group (Fig. 7G).

## Discussion

In recent decades, laryngeal squamous cell carcinoma (LSCC) is the most common malignancies in laryngeal cancer. Despite comprehensive therapeutic advances for LSCC have improved, the outcomes for patients with advanced LSCC have not improved in the last 30 years (Jenckel & Knecht, 2013). LSCC is prone to local and distant invasion, and lymph node metastasis, which are the main factors for poor prognosis (Gao et al., 2019). Therefore, discovering more effective biomarkers that can accurately signify the prognosis of patients would be essential for LSCC diagnosis and treatment.

The present study screened two gene expression profiles from GEO database. Using differential gene expression analysis, A total of 125 overlapping DEGs were found. These DEGs were enriched in the Human papillomavirus infection, cellular senescence, cell cycle and pathways in cancer. Also, the Cytoscape MCODE was used to screen network modules and identify hub genes. The hub genes were *MELK*, *MCM2*, *TPX2*, *FOXM1*, *CEP55*, *CHEK1*, *CDCA5*, *TK1* and *BIRC5*. The survival analysis showed that only the high expression of BIRC5 was associated with poor overall survival. *BIRC5*, also known as survivin, was a member of the inhibitor-of-apoptosis proteins family identified and characterized in 1997 (Ambrosini, Adida & Altieri, 1997). BIRC5 is located in the nucleus and regulated cell cycle-related factors. It is essential for mitotic checkpoint and participates in cell differentiation, proliferation and invasion (Wang et al., 2012; Yamamoto, Ngan & Monden, 2008; Zwerts et al., 2007). However, the significance of BIRC5 expression in the prognosis of LSCC remains unclear. This is the first study to evaluate BIRC5 as a potential predictive biomarker for LSCC.

We performed integrated bioinformatics analysis to explore the expression and prognostic value of BIRC5 in LSCC. Here, we found that the expression of BIRC5 was elevated in patients with LSCC by analyzing TCGA database. Kaplan–Meier plotter and multivariate cox regression analysis validated that the increased expression of BIRC5 was associated with overall survival in LSCC patients. With the data from UALCAN dataset showed that BIRC5 had higher expression levels in advanced LSCC tumor grade. The results of western blot showed that BIRC5 was notably higher in LSCC cells compared with the HOK cell (data not show), which was consistent with mRNA level. These results collectively elucidated that the expression of BIRC5 could be a predictive biomarker for the prognosis of LSCC.

Dysregulated BIRC5 have been linked to therapeutic resistance in stage II/III breast cancer (Hamy et al., 2016). Moreover, upregulated BIRC5 was associated with poor prognosis of ovarian cancer (Zhao et al., 2017). Dong et al. previously demonstrated that the expression of survivin is associated with unfavorable clinical pathological parameters and could serve as a biomarker for prognosis of LSCC patients (Dong et al., 2002). Another study by Pizem, Cor & Gale (2004) a high level of survivin expression predicts poor survival of LSCC. Consistently, we found that BIRC5 was tightly correlated with LSCC progression and tumorigenesis. Of note, our original prediction was based on TCGA and GEO datasets analyses, we found *BIRC5* was strongly correlated with higher tumor grade of LSCC. More importantly, our Immunohistochemical (IHC) results validated that BIRC5 was highly expressed in LSCC tissues when compared with normal tissues. RT-PCR, western blot, colony-formation assay, and flowcytometry were designed to reveal the relationship between BIRC5 expression and LSCC progression. Collectively, as observed in some other cancer types (Cao et al., 2019; Liu, Lin & He, 2019), our findings highlight the potential of BIRC5 as a novel biomarker for LSCC patients.

Although our current study have screened and validated the potential prognostic biomarker of LSCC, some limitations still exist. Such as the number of samples from the database is insufficient to avoid bias. The clinical characteristics of human tissues used in this study are incomplete. Our results might provide valuable information and pathways concerned with LSCC for better clarifying the molecular mechanism of LSCC carcinogenesis.

## Conclusions

In summary, the present study identified nine hub genes by data mining from TCGA and GEO datasets, and further analysis demonstrated that BIRC5 might be a prognostic biomarker in LSCC. Our findings revealed that BIRC5 could promote laryngeal cancer tumorigenesis and contribute to the poor prognosis of LSCC patients. BIRC5 may serve as an independent novel prognostic factor for LSCC.

## Supplemental Information

10.7717/peerj.12871/supp-1 Supplemental Information 1 Raw data for WB, RT-PCR, cell cycle and apoptosis.

10.7717/peerj.12871/supp-2 Supplemental Information 2 Upregulated DEGs of GSE51985 and GSE59102.

10.7717/peerj.12871/supp-3 Supplemental Information 3 Downregulated DEGs of GSE51985 and GSE59102.

10.7717/peerj.12871/supp-4 Supplemental Information 4 Clinical information.

## Additional Information and Declarations

### Data Availability

The following information was supplied regarding data availability: The raw data is available in the Supplemental Files and at GEO: GSE51985 and GSE59102.
