# Supplementary figures and images for: BIRC5 promotes cancer progression and predicts prognosis in laryngeal squamous cell carcinoma
Source: PeerJ. 2022 Feb 1;10:e12871. doi: 10.7717/peerj.12871 (PMC8815368; doi:10.7717/peerj.12871)

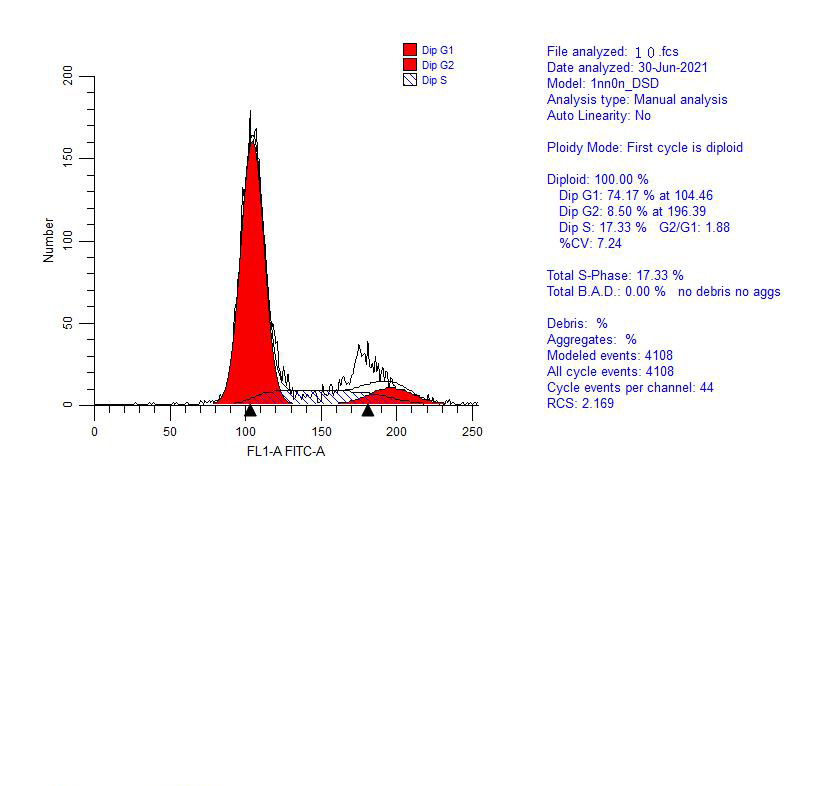

Supplement: Supplemental Information 1 [file peerj-10-12871-s001.zip › Raw data/rpt_ú▒ú░.fcs.jpg]

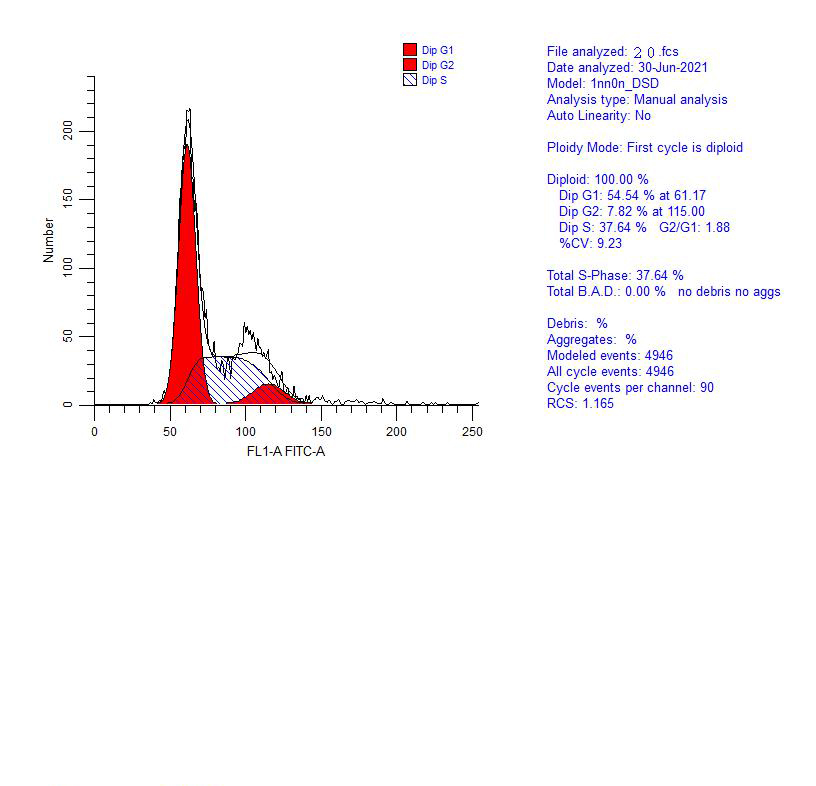

Supplement: Supplemental Information 1 [file peerj-10-12871-s001.zip › Raw data/rpt_ú▓ú░.fcs.jpg]

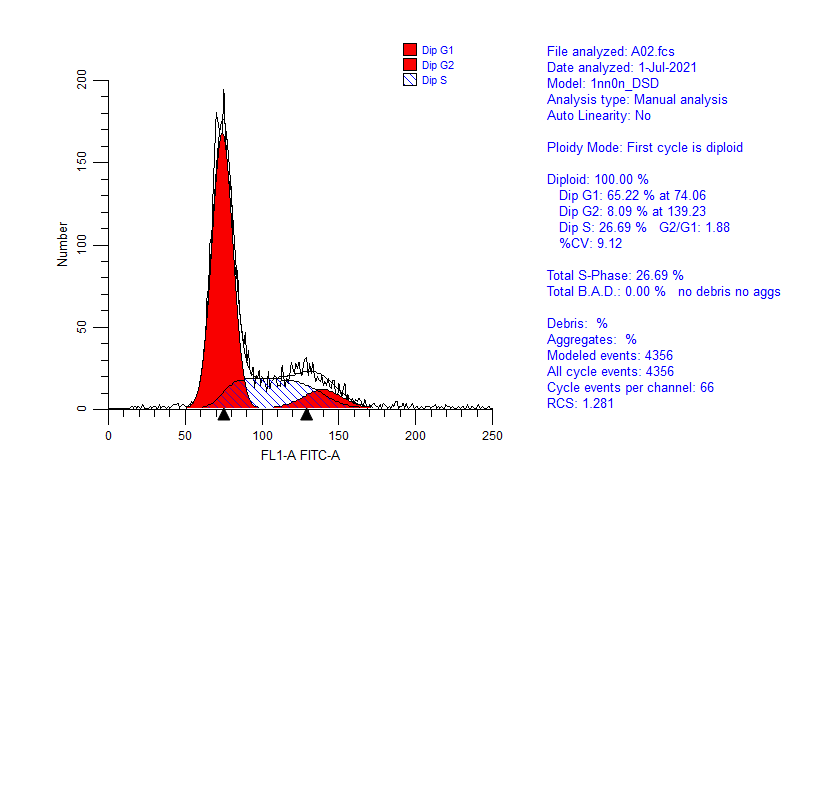

Supplement: Supplemental Information 1 [file peerj-10-12871-s001.zip › Raw data/rpt_4 0.fcs.tif]

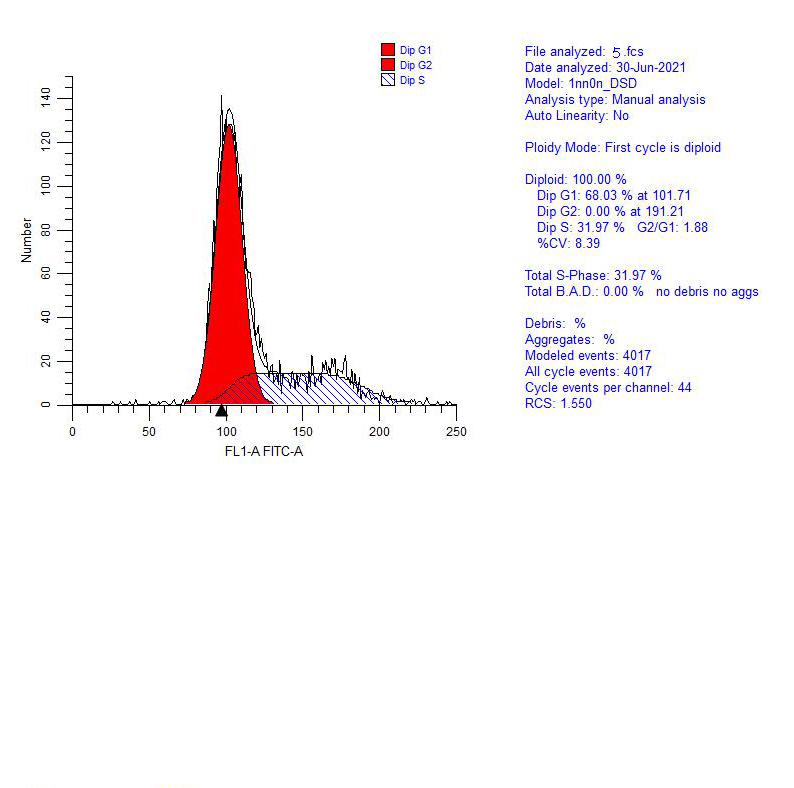

Supplement: Supplemental Information 1 [file peerj-10-12871-s001.zip › Raw data/rpt_ú╡.fcs1.jpg]

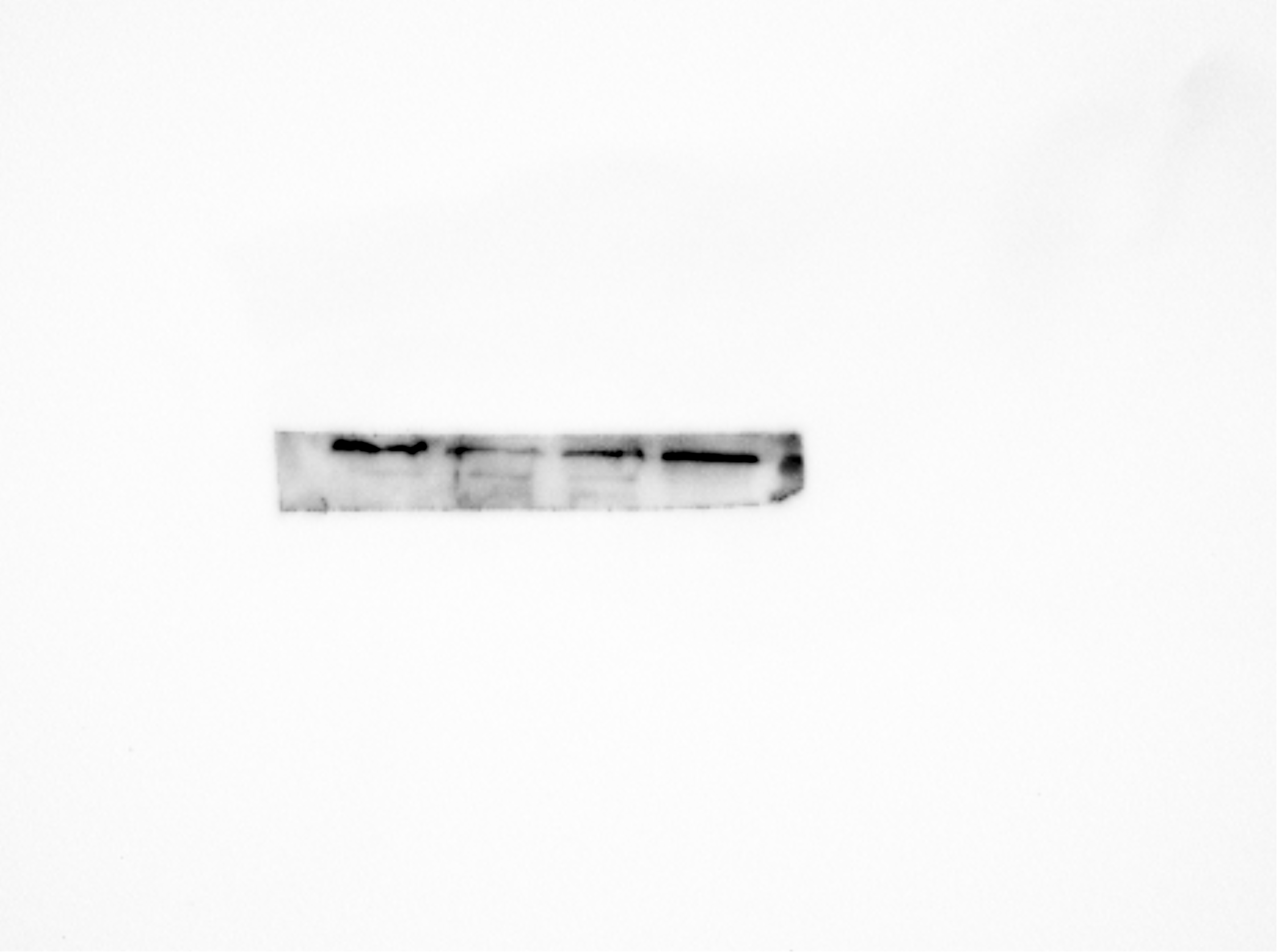

Supplement: Supplemental Information 1 [file peerj-10-12871-s001.zip › Raw data/wb/birc5 2.tif]

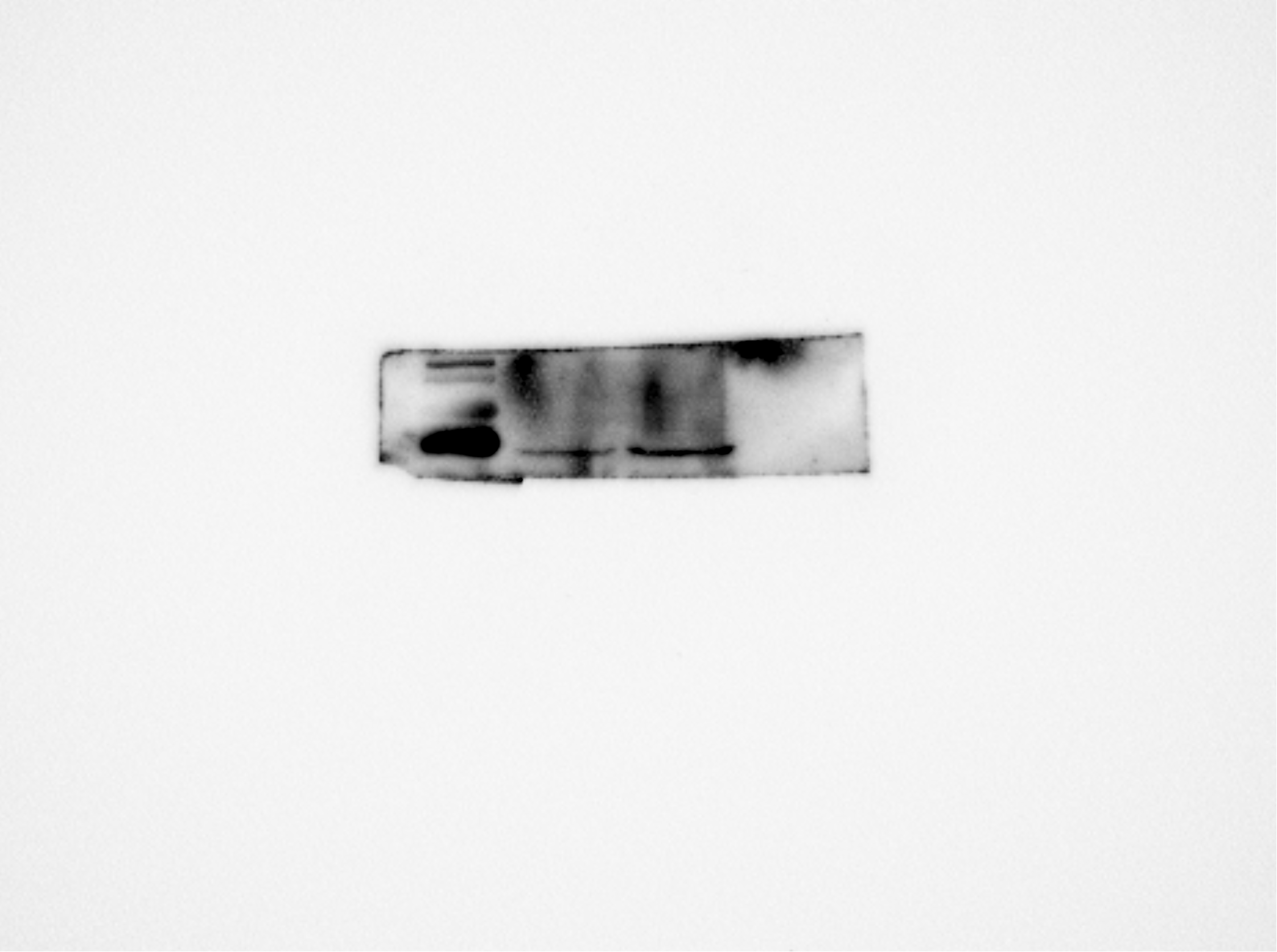

Supplement: Supplemental Information 1 [file peerj-10-12871-s001.zip › Raw data/wb/birc5.tif]

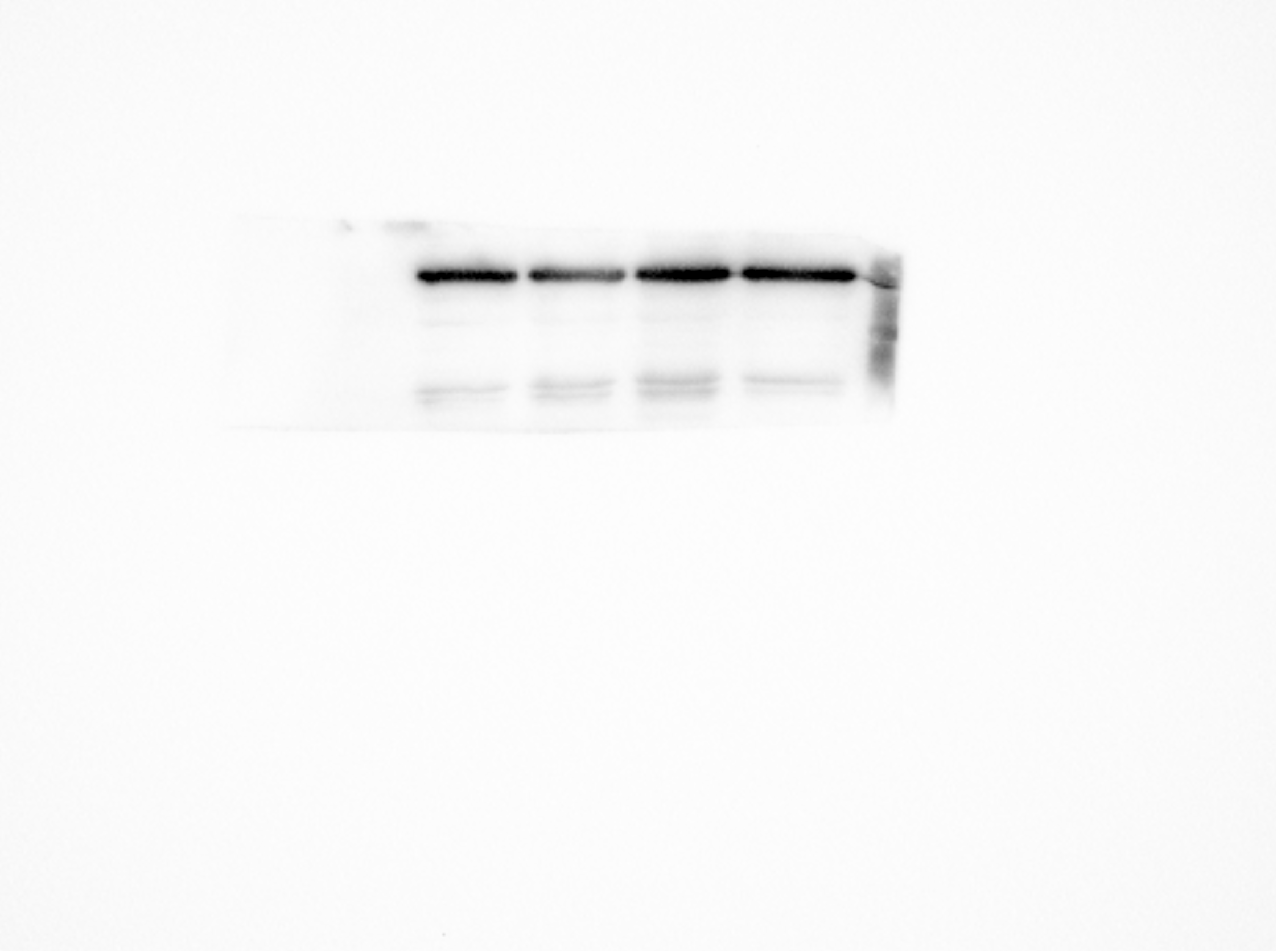

Supplement: Supplemental Information 1 [file peerj-10-12871-s001.zip › Raw data/wb/gapdh 2.tif]

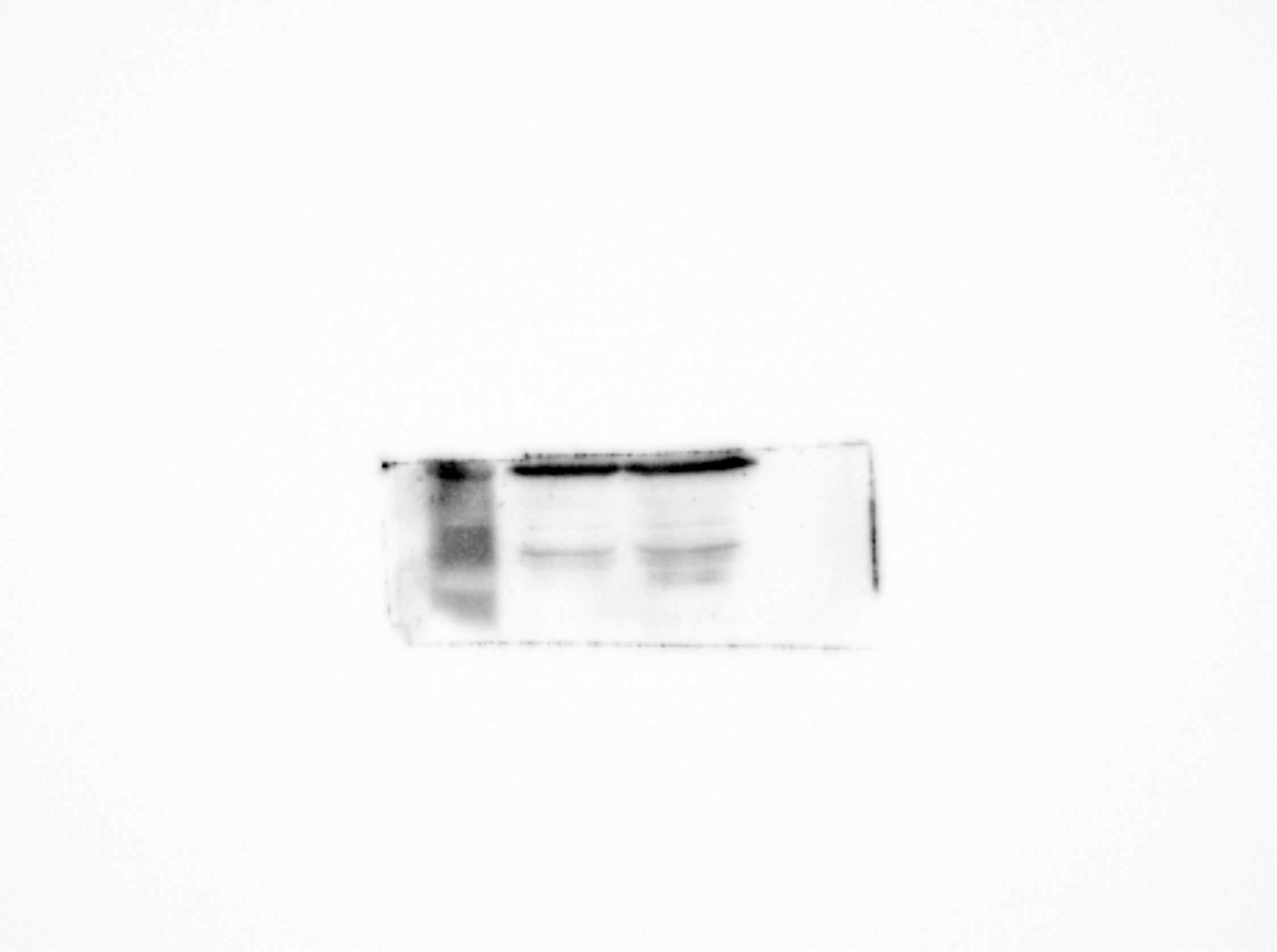

Supplement: Supplemental Information 1 [file peerj-10-12871-s001.zip › Raw data/wb/gapdh.tif]
