# Supplementary material for: BIRC5 promotes cancer progression and predicts prognosis in laryngeal squamous cell carcinoma
Source: PeerJ. 2022 Feb 1;10:e12871. doi: 10.7717/peerj.12871 (PMC8815368; doi:10.7717/peerj.12871)
Supplement: Supplemental Information 2 [file peerj-10-12871-s002.docx]

| Gene name | GSE51985 | | GSE59102 | |
| --- | --- | --- | --- | --- |
|  | logFC | P | logFC | P |
| APOC1 | 2.487827 | 1.49E-04 | 2.2 | 1.08E-05 |
| BIRC5 | 2.655332 | 6.12E-04 | 2.02 | 2.61E-09 |
| CDCA5 | 2.154699 | 2.26E-04 | 2.12 | 2.17E-11 |
| CEP55 | 3.167709 | 4.95E-03 | 2.39 | 6.01E-10 |
| CHEK1 | 2.11036 | 1.66E-03 | 2.13 | 1.29E-11 |
| COL1A1 | 2.399835 | 3.34E-03 | 2.38 | 8.99E-06 |
| CSF2 | 2.172401 | 2.52E-03 | 4.61 | 1.62E-11 |
| CTHRC1 | 2.394691 | 6.44E-04 | 2.2 | 9.25E-07 |
| FBN2 | 2.927495 | 3.02E-03 | 3.06 | 6.16E-06 |
| FOXM1 | 2.10046 | 1.63E-04 | 2.36 | 6.43E-10 |
| HOXC13 | 2.138952 | 1.27E-04 | 5.52 | 9.93E-15 |
| HOXD10 | 2.247164 | 8.58E-04 | 4.13 | 2.74E-13 |
| HOXD11 | 4.498622 | 9.44E-08 | 3.86 | 1.89E-13 |
| ISG15 | 2.084231 | 3.51E-04 | 3.18 | 2.27E-12 |
| KRT14 | 2.177323 | 3.47E-03 | 2.78 | 8.28E-08 |
| KRT16 | 3.583999 | 3.58E-06 | 4.1 | 8.24E-11 |
| KRT16P2 | 3.227339 | 9.55E-04 | 2.9 | 3.73E-09 |
| KRT17 | 2.679858 | 1.02E-06 | 3.05 | 1.10E-08 |
| KRT6B | 2.74714 | 5.36E-05 | 2.45 | 1.79E-04 |
| LAMA3 | 2.266854 | 5.59E-05 | 2.62 | 2.50E-08 |
| LAMC2 | 2.606734 | 1.77E-04 | 3.35 | 8.51E-12 |
| MCM2 | 2.054654 | 5.60E-05 | 2.23 | 1.48E-14 |
| MELK | 2.211047 | 1.25E-03 | 2.02 | 4.88E-09 |
| MMP10 | 3.369248 | 1.02E-03 | 3.03 | 3.50E-03 |
| NELL2 | 2.05837 | 4.67E-03 | 2.94 | 3.36E-06 |
| NRG1 | 2.479725 | 1.41E-04 | 2.27 | 6.31E-07 |
| OASL | 2.799061 | 1.93E-03 | 3.33 | 6.98E-11 |
| PLAU | 2.444088 | 1.17E-04 | 2.28 | 5.67E-08 |
| PPP1R14C | 2.036348 | 5.36E-04 | 2.39 | 7.65E-14 |
| PTHLH | 4.243699 | 3.73E-05 | 4.27 | 2.75E-08 |
| RASL11B | 2.12548 | 5.66E-04 | 2.68 | 1.32E-09 |
| SERPINE1 | 2.307746 | 1.10E-03 | 3.97 | 5.36E-09 |
| SLC52A1 | 2.056665 | 5.26E-05 | 2.1 | 1.74E-07 |
| SPP1 | 3.415011 | 5.39E-06 | 3.74 | 6.55E-09 |
| STC2 | 2.451755 | 1.94E-04 | 2.3 | 2.11E-06 |
| TK1 | 2.073475 | 5.09E-04 | 2.11 | 1.20E-12 |
| TMEM158 | 2.613058 | 2.41E-05 | 2.49 | 9.92E-09 |
| TNC | 2.241039 | 1.14E-04 | 2.04 | 5.53E-04 |
| TNFRSF12A | 2.28629 | 2.23E-03 | 2.67 | 7.73E-08 |
| TPX2 | 2.269009 | 2.51E-04 | 2.28 | 1.93E-11 |
| WDR66 | 2.640017 | 2.39E-04 | 3.43 | 1.65E-07 |
