# Supplementary material for: BIRC5 promotes cancer progression and predicts prognosis in laryngeal squamous cell carcinoma
Source: PeerJ. 2022 Feb 1;10:e12871. doi: 10.7717/peerj.12871 (PMC8815368; doi:10.7717/peerj.12871)
Supplement: Supplemental Information 3 [file peerj-10-12871-s003.docx]

| Gene name | GSE51985 | | GSE59102 | |
| --- | --- | --- | --- | --- |
|  | logFC | P | logFC | P |
| ABCA8 | -2.81582 | 2.52E-07 | -3.39 | 4.79E-09 |
| ACKR1 | -2.12326 | 5.86E-04 | -2.03 | 5.02E-05 |
| ACOX2 | -2.33212 | 2.07E-06 | -2.58 | 7.86E-12 |
| ADGRF1 | -2.03983 | 1.97E-03 | -3.51 | 9.48E-06 |
| ADH1A | -3.19975 | 5.08E-04 | -3.2 | 1.68E-04 |
| AGR2 | -2.04783 | 7.29E-03 | -2.98 | 6.04E-04 |
| AGR3 | -2.73529 | 2.06E-03 | -4.51 | 1.10E-07 |
| ALDH1L1 | -3.51268 | 1.88E-06 | -2.65 | 1.39E-05 |
| APOD | -2.67514 | 1.93E-04 | -3.72 | 1.17E-07 |
| B3GNT6 | -2.49157 | 6.26E-04 | -3 | 7.63E-08 |
| BCAS1 | -2.61788 | 4.17E-05 | -3.37 | 1.47E-08 |
| CCL14 | -3.00763 | 1.01E-04 | -3.6 | 4.42E-08 |
| CEACAM1 | -2.32416 | 2.04E-03 | -3.02 | 2.37E-06 |
| CFD | -2.14964 | 5.81E-07 | -2.47 | 2.44E-08 |
| CGNL1 | -2.98358 | 1.06E-03 | -3.39 | 4.04E-09 |
| CH25H | -2.21059 | 1.68E-04 | -2.34 | 1.27E-05 |
| CILP | -2.11374 | 1.73E-03 | -3.68 | 7.07E-11 |
| CLDN10 | -3.30728 | 1.41E-04 | -4.59 | 3.76E-06 |
| CLEC3B | -2.55138 | 1.89E-08 | -3.14 | 5.97E-12 |
| COBL | -2.06263 | 2.42E-05 | -2.65 | 1.07E-05 |
| CXCL12 | -3.1692 | 4.98E-06 | -4.12 | 1.52E-08 |
| CXCL17 | -2.40906 | 5.06E-04 | -4.14 | 1.55E-06 |
| CYP2J2 | -2.12251 | 1.27E-04 | -2.57 | 1.35E-09 |
| CYP4B1 | -4.51067 | 8.76E-05 | -4.47 | 8.52E-09 |
| DNALI1 | -2.05167 | 4.23E-03 | -2.18 | 2.46E-05 |
| EBF1 | -2.02553 | 8.08E-04 | -2.09 | 6.82E-11 |
| FAM107A | -3.43841 | 1.17E-06 | -3.48 | 1.15E-12 |
| FAM174B | -2.83279 | 1.17E-05 | -2.1 | 1.94E-09 |
| FAM3B | -3.08778 | 5.29E-05 | -3.92 | 8.29E-07 |
| FAM3D | -3.70576 | 2.22E-05 | -5.08 | 1.90E-10 |
| FOLR1 | -4.09929 | 2.79E-04 | -2.89 | 2.61E-05 |
| FRZB | -2.07867 | 2.17E-03 | -2.47 | 9.76E-06 |
| GABRP | -3.61429 | 3.65E-03 | -3.8 | 1.37E-07 |
| GCNT3 | -2.97177 | 3.67E-05 | -3.28 | 6.82E-08 |
| GNG7 | -2.07603 | 5.88E-04 | -2.05 | 2.45E-06 |
| KBTBD11 | -2.16017 | 1.70E-05 | -2.07 | 1.10E-07 |
| LEPR | -2.52205 | 1.01E-05 | -2.95 | 3.83E-09 |
| LTF | -3.19259 | 1.20E-03 | -4.42 | 8.91E-04 |
| LYZ | -2.92136 | 5.93E-03 | -4.03 | 2.11E-05 |
| MAATS1 | -3.14761 | 2.96E-03 | -2.6 | 1.83E-06 |
| MFAP4 | -2.08815 | 2.39E-05 | -2.32 | 1.14E-06 |
| MIR99AHG | -2.28531 | 8.26E-06 | -2.02 | 6.97E-06 |
| MLPH | -2.14783 | 4.83E-03 | -3.36 | 1.93E-07 |
| MMRN1 | -3.57315 | 1.34E-03 | -3.01 | 1.62E-07 |
| MUC1 | -2.30102 | 7.05E-05 | -2.1 | 8.34E-07 |
| NBEA | -2.16955 | 4.65E-04 | -3.09 | 1.88E-08 |
| NKX3-1 | -2.05319 | 6.85E-03 | -2.17 | 4.58E-05 |
| NOSTRIN | -2.05546 | 2.23E-05 | -2.24 | 1.03E-09 |
| NR3C2 | -2.12944 | 1.00E-04 | -2.76 | 1.81E-09 |
| OXGR1 | -2.41413 | 2.36E-03 | -3.91 | 9.25E-09 |
| PDK4 | -2.70493 | 8.00E-04 | -3.09 | 5.35E-10 |
| PGM5 | -2.81402 | 1.48E-05 | -2.96 | 6.43E-10 |
| PIP | -3.93731 | 1.36E-03 | -4.51 | 6.99E-06 |
| PLEKHB1 | -2.60146 | 3.72E-04 | -2.33 | 2.39E-05 |
| PPP1R3C | -2.90299 | 6.27E-05 | -2.92 | 5.64E-06 |
| PRB1 | -5.30088 | 5.57E-04 | -5.21 | 3.33E-06 |
| PRB3 | -5.06089 | 5.71E-04 | -4.08 | 9.85E-07 |
| PRR15L | -2.24201 | 5.44E-05 | -3.51 | 4.19E-07 |
| PTGDS | -2.88885 | 2.07E-04 | -3.05 | 7.03E-07 |
| PTPRN2 | -2.3033 | 8.48E-05 | -2.55 | 3.67E-08 |
| RNASE4 | -2.4231 | 2.30E-05 | -2.53 | 7.91E-09 |
| SCGB3A2 | -3.61163 | 4.73E-03 | -3.45 | 1.97E-05 |
| SCIN | -2.65434 | 9.32E-03 | -3.59 | 1.05E-07 |
| SELENBP1 | -2.63685 | 1.58E-04 | -2.85 | 8.21E-09 |
| SERPINB11 | -2.2426 | 8.15E-03 | -2.62 | 4.30E-06 |
| SFRP1 | -2.81169 | 4.43E-05 | -2.49 | 4.34E-04 |
| SH3BGRL2 | -2.83191 | 1.81E-06 | -4.01 | 1.33E-12 |
| SLC44A3 | -2.00995 | 1.14E-04 | -2.39 | 1.43E-10 |
| SLIT3 | -2.13552 | 5.91E-06 | -2.02 | 7.07E-08 |
| SLITRK5 | -2.02865 | 4.44E-03 | -2.08 | 2.06E-07 |
| SLPI | -2.02302 | 1.21E-03 | -2.26 | 4.45E-05 |
| SORBS2 | -3.10341 | 5.41E-09 | -3.16 | 1.04E-13 |
| SPATA18 | -2.18545 | 6.81E-04 | -2.56 | 8.62E-10 |
| SPDEF | -2.8156 | 3.75E-03 | -2.36 | 2.16E-06 |
| ST6GALNAC1 | -2.70576 | 1.56E-05 | -3.07 | 3.03E-06 |
| STATH | -4.56396 | 2.45E-04 | -6.84 | 5.91E-08 |
| TCN1 | -2.58448 | 3.60E-03 | -4.51 | 2.05E-06 |
| TF | -2.53485 | 8.76E-04 | -4.29 | 4.12E-07 |
| TFF3 | -3.41029 | 2.55E-04 | -5.07 | 2.45E-08 |
| TMEM100 | -2.46472 | 2.40E-05 | -2.92 | 4.27E-11 |
| TMPRSS2 | -2.80556 | 2.50E-03 | -2.22 | 1.00E-07 |
| TSPAN8 | -2.84364 | 7.09E-04 | -3.9 | 5.70E-06 |
| VSIG2 | -3.39463 | 8.96E-06 | -2.88 | 6.09E-11 |
| ZG16B | -4.68385 | 7.92E-05 | -5.07 | 3.19E-06 |
